# The Methyltransferase *VdPRMT4* Regulates *Verticillium dahliae* via Regulation of Primary Metabolic Processes

**DOI:** 10.3390/jof12050369

**Published:** 2026-05-16

**Authors:** Yanqing Bi, Guoshuai Zhang, Xinyu Zhu, Yumei Su, W. G. Dilantha Fernando, Xiaofeng Su, Wenfang Guo, Yue Li

**Affiliations:** 1 Key Laboratory of Biological Ecological Adaptation and Evolution in Extreme Environments, College of Life Science, Xinjiang Agricultural University, Urumqi 830052, China; b916228271@163.com (Y.B.); 17325221793@163.com (G.Z.); 19922614126@163.com (X.Z.); suyumeicaas@163.com (Y.S.); 2 Department of Plant Science, University of Manitoba, Winnipeg, MB R3T 2N2, Canada; dilantha.fernando@umanitoba.ca; 3 St. Paul’s College, University of Manitoba, Winnipeg, MB R3T 2M6, Canada; 4 National Key Laboratory of Agricultural Microbiology, Biotechnology Research Institute, Chinese Academy of Agricultural Sciences, Beijing 100081, China; suxiaofeng@caas.cn

**Keywords:** verticillium wilt, *V. dahliae*, *VdPRMT4*, PRMT

## Abstract

Cotton Verticillium wilt (VW), caused by the soil-borne fungus *Verticillium dahliae* (*V. dahliae*), is a devastating disease that poses a serious threat to sustainable cotton production worldwide. Protein methylation plays a critical role in fungal adaptation to the host environment and manipulation of plant immunity. Protein arginine methyltransferases (PRMTs) are key enzymes catalyzing arginine methylation, yet their functions in *V. dahliae* pathogenicity remain largely unexplored. In this study, we identified *VdPRMT4* in *V. dahliae* through homology-based screening. qRT-PCR analysis revealed that *VdPRMT4* transcript levels were significantly increased during the early stages of *V. dahliae* infection in cotton. HIGS assays showed that silencing *VdPRMT4* markedly alleviated cotton VW symptoms and reduced fungal biomass in cotton plants. Gene knockout and complementation experiments demonstrated that deletion of *VdPRMT4* did not affect hyphal growth but significantly impaired sporulation capacity and severely attenuated pathogenicity on cotton. Transcriptomic analysis further indicated that loss of *VdPRMT4* profoundly affected the metabolic pathways of *V. dahliae*, including protein processing in the endoplasmic reticulum, purine metabolism, and glycerolipid metabolism. Collectively, this study provides the first evidence that *VdPRMT4* plays a critical role in stress adaptation and pathogenicity of *V. dahliae*, offering new insights into fungal pathogenesis and identifying potential targets for VW control.

## 1. Introduction

Cotton Verticillium wilt (VW), caused by the soil-borne pathogen *Verticillium dahliae*, is one of the most destructive diseases affecting cotton production worldwide, leading to significant yield losses and deterioration of fiber quality. The pathogen can persist in soil for long periods as microsclerotia, invade host roots, colonize the xylem, and ultimately induce systemic wilting and plant death [1]. While research on traditional virulence factors, such as cell wall-degrading enzymes and effectors, is well advanced, recent studies indicate that epigenetic regulation plays a crucial role in the pathogen’s adaptation to the host environment and in manipulating plant immunity [2]. Epigenetic regulation, including DNA methylation and histone modifications, modulates gene expression without altering the DNA sequence [3,4]. In fungal pathogens, these reversible modifications enable rapid transcriptional reprogramming in response to host environmental cues, thereby influencing developmental transitions, stress adaptation, and virulence [2].

Among these diverse epigenetic mechanisms, protein methylation, particularly arginine methylation catalyzed by protein arginine methyltransferases (PRMTs), constitutes a critical and conserved regulatory layer in eukaryotes [5]. PRMTs catalyze the transfer of methyl groups to arginine residues, generating mono- or dimethylated derivatives that can profoundly alter protein function, stability, and interaction networks [6]. PRMT family members are classified into different subfamilies based on their catalytic activities and substrate specificities. These enzymes share a conserved methyltransferase domain responsible for catalysis, while their N-terminal and C-terminal regions confer substrate recognition and functional specificity [7,8,9]. Beyond histone methylation, PRMTs modify a wide array of non-histone substrates involved in RNA splicing and signal transduction, thereby playing pivotal roles in diverse biological processes across eukaryotes [10].

In plant pathogenic fungi, the functions of PRMTs are gradually being elucidated, pointing to their potential importance in pathogenicity. In *Magnaporthe oryzae*, PRMT family members, including MoHMT1, have been shown to regulate conidiation, appressorium formation, and infectious growth [11]. In *Penicillium expansum*, PeRmtC directly affects development and pathogenicity by regulating key genes involved in conidiation and secondary metabolism [12]. Furthermore, in the human pathogen *Candida albicans*, arginine methylation participates in morphological switching and virulence regulation [13]. This cross-species evidence suggests a conserved theme in which arginine methylation acts as a key epigenetic switch regulating fungal development and pathogenicity. In the oomycete pathogen *Phytophthora capsici*, deletion of the type I PRMT gene *PcPRMT3* significantly affected mycelial growth, asexual spore development, pathogenicity, and stress responses, with transcriptome analyses indicating disruption of multiple biological pathways and increased susceptibility to oxidative stress [14]. However, despite this progress, the specific roles and regulatory networks of arginine methyltransferases in *V. dahliae* remain largely unexplored, presenting a significant gap in understanding the epigenetic underpinnings of its virulence. To date, only a single study by Wu et al. has provided preliminary insights, demonstrating that the protein arginine methyltransferase *VdPRMT1* is involved in regulating vegetative growth and conidiation in *V. dahliae* and that deletion of *VdPRMT1* results in a significant reduction in pathogenicity on cotton [15]. However, the functions and regulatory mechanisms of other PRMT family members in this fungus remain entirely unknown. Therefore, systematically elucidating the specific roles and regulatory networks of arginine methyltransferases in *V. dahliae* is of great scientific significance for understanding the epigenetic basis of pathogenicity in this destructive pathogen.

Building upon the emerging understanding of epigenetic regulation in fungal pathogens, particularly the conserved role of arginine methylation, we next consider how such regulatory mechanisms may integrate with the physiological adaptability of the pathogen—a key determinant of successful infection. The pathogenicity of *V. dahliae* is closely associated with its ability to adapt to environmental stresses and its reproductive strategies. Research indicates that the pathogen’s efficiency in carbon source utilization, tolerance to cell wall stresses (e.g., Congo Red, calcofluor white), oxidative stress (e.g., H_2_O_2_), and high salinity directly affect its colonization and reproductive success [16,17,18,19]. For example, the mating-type genes *VdMAT1-1-1* and *VdMAT1-2-1* have been shown to regulate carbon source utilization, stress tolerance, and conidial production [20]. The 24 kDa subunit of mitochondrial complex I simultaneously influences mycelial growth, microsclerotia development, stress tolerance, and virulence [21]. Moreover, deletion of carbon catabolite repression (CCR)-related genes *VdCreA* and *VdCreC* leads to slow growth, decreased sporulation, and reduced virulence, highlighting the direct link between carbon metabolism and pathogenicity [22]. Collectively, these studies demonstrate that the physiological adaptability of the pathogen is fundamental to successful infection.

Transcriptomic analyses of fungal pathogens have revealed that infection extensively affects host metabolic pathways. Among these, protein processing in the endoplasmic reticulum (ER) is frequently targeted by fungal pathogens, with unfolded protein response (UPR) signaling and N-glycosylation-related ER proteins essential for host penetration, colonization, and effector secretion in diverse pathogens such as *Magnaporthe oryzae* and *Colletotrichum graminicola* [23]. Purine metabolism plays a fundamental role in fungal growth and pathogenicity, as blockade of this pathway inhibits penetration structure formation and invasive growth in *Magnaporthe oryzae* and *Fusarium graminearum*, rendering it essential for plant infection [24]. Glycerolipid metabolism, which provides precursors for defense signaling molecules, also influences virulence—disruption of glycerolipid homeostasis in *Candida albicans* limits hyphal induction and reduces virulence [25], whereas in *Setosphaeria turcica*, lipid metabolism contributes to glycerol accumulation in appressoria [26]. The convergence of these three metabolic pathways highlights how pathogens modulate host primary metabolism to create a favorable microenvironment for colonization. Investigating whether *VdPRMT4* influences these interconnected pathways may provide insights into its potential role in fungal pathogenicity.

Against this background, this study employed an integrated approach combining bioinformatics, expression analysis, host-induced gene silencing (HIGS), gene knockout and complementation, phenotypic analysis under multiple stresses, and comparative transcriptomics to systematically elucidate the function of *VdPRMT4* in *V. dahliae*. The research identified *VdPRMT4* as a potential virulence determinant; its deletion did not affect basal hyphal growth but significantly altered the pathogen’s adaptability for sporulation under stress conditions and severely attenuated its pathogenicity on cotton. Comparative transcriptome analysis of PDA-grown cultures showed that deletion of *VdPRMT4* led to significant alterations in the expression of genes in several pathways within *V. dahliae*, including protein processing in the endoplasmic reticulum, purine metabolism, and glycerolipid metabolism. This study provides the first systematic demonstration that *VdPRMT4* functions as a novel epigenetic regulator in *V. dahliae*, offering new insights into the pathogenic mechanisms of fungal pathogens and the development of novel control strategies.

## 2. Materials and Methods

### 2.1. Plant and Fungal Materials and Growth Conditions

The upland cotton (*Gossypium hirsutum*) cultivar ‘Zhongmian 49’ was used in this study. Seeds were surface-sterilized with 75% (*v*/*v*) ethanol for 30 s, rinsed thoroughly with sterile water, and sown in a mixed substrate of vermiculite and nutrient soil (1:2, *v*/*v*). Seedlings were grown in a controlled growth chamber at 25 °C, 70% of relative humidity, and under a 16-h light/8-h dark photoperiod. The highly virulent defoliating pathotype strain *Vd*991 of *V. dahliae* was kindly provided by the Laboratory of Crop Functional Genomics and Molecular Improvement, College of Life Sciences, Xinjiang Agricultural University. For routine culture and inoculum preparation, the fungus was grown on potato dextrose agar (PDA) at 25 °C in the dark. To prepare spore suspensions for inoculation, mycelial plugs were transferred to complete liquid medium and incubated at 25 °C with shaking at 200 rpm for 5 days. The culture was filtered through four layers of sterile cheesecloth to remove mycelia. Conidia in the filtrate were counted using a hemocytometer and adjusted to a final concentration of 1 × 10^7^ colony-forming units (CFU/mL) with sterile water.

### 2.2. Cloning and Bioinformatic Analysis of VdPRMT4

Specific primers for amplifying the *VdPRMT4* gene were designed based on its annotated sequence in the *V. dahliae* genome database using Primer Premier 5 software (Premier Biosoft, San Francisco, CA, USA). The target fragment was amplified from cDNA using a high-fidelity DNA polymerase, verified by agarose gel electrophoresis, purified with the Gel Extraction Kit (Tiangen, Beijing, China), and cloned into the pEASY-Blunt Zero vector for sequencing. The molecular weight and isoelectric point of the predicted protein were analyzed using the ExPASy ProtParam tool (https://swissmodel.expasy.org/) (accessed on 1 February 2025). Homologous protein sequences of PRMTs from different species were retrieved from the NCBI database. A phylogenetic tree was constructed using the neighbor-joining method in MEGA11 software (Sudhir Kumar, Philadelphia, PA, USA). Conserved motifs were analyzed using the MEME online tool (https://meme-suite.org/meme/) (accessed on 1 August 2025). Multiple sequence alignment was performed and visualized using ESPript 3.0 (https://espript.ibcp.fr/ESPript/ESPript/) (accessed on 1 February 2025), and protein domains were predicted using the SMART online tool (https://smart.embl.de/smart/change_mode.cgi) (accessed on 1 February 2025).

### 2.3. Gene Expression Pattern Analysis

To determine whether *VdPRMT4* is involved in infection of cotton by *V. dahliae*, we examined its expression in the fungus at different time points (0, 2, 4, 8, 12, 24, 48, 96, 120, and 144 h post-inoculation, hpi) using qRT-PCR. Total RNA was extracted from inoculated cotton root tissues using the RNAprep Pure Plant Kit (Tiangen, Beijing, China). First-strand cDNA was synthesized from 1 µg of total RNA using the HiScript IV All-in-One Ultra RT SuperMix (Vazyme, Nanjing, China) for qRT-PCR. qRT-PCR was performed using ChamQ Universal SYBR qPCR Master Mix (Vazyme, Nanjing, China) on an ABI 7500 Fast Real-Time PCR System (Applied Biosystems, Foster City, CA, USA). The reaction program was as follows: 95 °C for 30 s, followed by 40 cycles of 95 °C for 10 s and 60 °C for 30 s, ending with a melt curve analysis. Three biological replicates and three technical replicates were included for each sample. The relative expression level was calculated using the 2^−ΔΔCt^ method, with the *V. dahliae* actin gene (*VDAG_02600*) serving as the internal reference.

### 2.4. HIGS Vector Construction and Inoculation

To construct the host-induced gene silencing (HIGS) vector, a specific fragment of approximately 300 bp of the *VdPRMT4* gene was amplified by PCR using the corresponding sequence as template. The fragment was digested and ligated into the multiple cloning site of the pTRV2 vector to generate the recombinant plasmid pTRV2:*VdPRMT4*. The *GhCLA1* served as a positive control, and the empty pTRV2 vector was used as a negative control. All plasmids were separately introduced into *Agrobacterium tumefaciens* strain GV3101 competent cells using the heat shock method. Positive single colonies were cultured in LB liquid medium containing 50 µg/mL kanamycin and 50 µg/mL rifampicin until the OD_600_ reached 0.6–0.8. Bacterial cells were collected by centrifugation and resuspended in infiltration buffer to an OD_600_ of 1.0. For agroinfiltration, equal volumes of the pTRV1 culture and each pTRV2 recombinant culture were mixed and injected into the fully expanded cotyledons of cotton seedlings. Inoculated plants were kept in darkness under high humidity for 24 h before being returned to normal growth conditions. Approximately 10–14 days later, when the positive control plants (pTRV2::*GhCLA1*) showed an obvious albino phenotype, the HIGS system was considered successfully established. Uniformly grown plants were then selected and inoculated with the prepared *Vd*991 spore suspension using the root wounding and dipping method.

### 2.5. Generation of VdPRMT4 Knockout Mutants

The knockout mutant was generated using *Agrobacterium tumefaciens*-mediated transformation (ATMT). Briefly, approximately 1 kb upstream and downstream flanking sequences of the *VdPRMT4* gene, along with a hygromycin B phosphotransferase cassette, were amplified from the wild-type (WT) *V. dahliae* strain *Vd*991. These fragments were fused by overlap extension PCR to assemble the knockout construct, which was then cloned into the binary vector pGKO, yielding pGKO-*ΔVdPRMT4*. This plasmid was introduced into *A. tumefaciens* strain EHA105.

For fungal transformation, conidia of *Vd*991 were co-cultivated with the *A. tumefaciens* suspension on IM solid medium overlaid with a filter membrane. After 48 h of co-cultivation at 25 °C in darkness, the membrane was transferred to PDA selection medium containing carbenicillin, cefotaxime, hygromycin B, and 5-fluorouracil to inhibit *A. tumefaciens* growth and select for transformants. Resistant colonies were purified through multiple rounds of subculture on selective PDA plates. Putative knockout mutants were verified by PCR using primers flanking the target site and primers internal to the hygromycin resistance gene. Two independent knockout mutants, designated *ΔVdPRMT4-1* and *ΔVdPRMT4-2*, were obtained and used for subsequent analyses.

### 2.6. Complementation of VdPRMT4 Knockout Mutants

Complementation strains were generated to confirm that the observed phenotypes were due to the loss of *VdPRMT4*. The full-length coding sequence (without the stop codon) of *VdPRMT4* was amplified from the WT strain and cloned into the binary vector pMC-*GFP*-G418, which carries a C-terminal GFP tag and a geneticin resistance marker, resulting in pMC-*VdPRMT4*-*GFP*.

The complementation vector was introduced into *A. tumefaciens* EHA105 and subsequently used to transform conidia of the *ΔVdPRMT4-1* and *ΔVdPRMT4-2* mutants via ATMT, following a similar co-cultivation and selection procedure as described above. Selection was performed on PDA medium supplemented with cefotaxime, ampicillin, and G418. Resistant colonies were purified and verified by PCR for the presence of the complemented gene. Expression of the VdPRMT4-GFP fusion protein was confirmed by observing GFP fluorescence under a fluorescence microscope. One complemented strain from each mutant background, designated *ΔVdPRMT4-1-C* and *ΔVdPRMT4-2-C*, was obtained for further study.

### 2.7. Phenotypic Analysis Under Different Carbon Sources and Stress Conditions

To assess carbon source utilization and stress tolerance, mycelial plugs of the WT, knockout mutant, and complemented strains were point-inoculated onto different solid media. Carbon source utilization tests were performed using basal medium supplemented with pectin (1%, *w*/*v*), galactose (1%, *w*/*v*), starch (1.4%, *w*/*v*), xylose (1%, *w*/*v*), or sucrose (3%, *w*/*v*) as the sole carbon source. Stress tolerance tests were conducted on PDA amended with the following agents: Congo Red (7.5%, *w*/*v*), calcofluor white (3%, *w*/*v*), SDS (0.01%, *w*/*v*), NaCl (4.095%, *w*/*v*), sorbitol (18.271%, *w*/*v*), or H_2_O_2_ (0.051%, *w*/*v*). All plates were incubated upside down at 25 °C in darkness for 14 days. Colony diameters were measured every two days, and the final average was calculated. After incubation, 2 mL of sterile water was added to each plate, and spores were gently scraped off, filtered through cheesecloth, and counted using a hemocytometer.

### 2.8. Pathogenicity Assay and Disease Evaluation

Pathogenicity was assessed using the root wounding and dipping method. Cotton seedlings at the two-true-leaf stage were carefully uprooted, their roots washed, and then wounded with sterile scissors before being immersed in a spore suspension (1 × 10^7^ CFU/mL) of each strain for 5 min. Seedlings immersed in sterile water served as the mock control. Inoculated plants were transplanted back into the substrate and maintained under standard growth conditions. At 14 days post-inoculation (dpi), disease severity was evaluated based on leaf wilting and yellowing symptoms according to the Chinese National Standard GB/T 22101.5-2009 [27]. A 0–4 grading scale was used: 0, no symptoms; 1, 1–25% of leaves affected; 2, 26–50%; 3, 51–75%; 4, 76–100% or plant death. The disease index was calculated using the following formula: DI = [Σ (disease grade × number of plants at that grade)/(total number of plants investigated × highest disease grade)] × 100. Simultaneously, root tissues were collected. Total DNA was extracted using a Plant Genomic DNA Kit, and the relative fungal biomass within the host was estimated by qRT-PCR, quantifying the copy number ratio of the pathogen rDNA gene (NR_126124.1) to the cotton reference gene *GhUBQ7* (LOC107912293).

### 2.9. Transcriptome Sequencing and Bioinformatics Analysis

The WT *Vd*991 and the *ΔVdPRMT4* mutant were cultured on PDA medium at 26 °C in the dark for 14 days, and samples were collected with three biological replicates per treatment. Samples were flash-frozen in liquid nitrogen and stored at −80 °C. Paired-end sequencing was performed on an Illumina NovaSeq 6000 platform. Raw sequencing reads were quality-filtered using Trimmomatic software (version 0.39) to remove low-quality reads and adapter sequences. High-quality clean reads were aligned to the *V. dahliae* (https://www.ncbi.nlm.nih.gov/datasets/genome/GCF_000150675.1, accessed on 19 May 2025) reference genome using HISAT2. Transcript assembly and gene expression quantification were performed using StringTie. Differential expression analysis was conducted using the DESeq2 R package (version 1.42.0), with genes having an absolute |log_2_FoldChange| > 1 and an adjusted *p*-value < 0.05 considered differentially expressed. Gene Ontology (GO) and Kyoto Encyclopedia of Genes and Genomes (KEGG) pathway enrichment analyses of the differentially expressed genes (DEGs) were performed using the ClusterProfiler R package (version 4.12.6). A protein–protein interaction (PPI) network was predicted using the STRING database (version 12.0; https://string-db.org) with a combined score threshold of >0.4 to identify high-confidence interactions among the proteins encoded by the DEGs. The resulting interaction data were imported into Cytoscape (version 3.9.1) for network visualization and analysis. To validate the reliability of the transcriptome sequencing results, 19 DEGs selected based on KEGG pathway analysis were verified using qRT-PCR.

### 2.10. Data Analysis

All phenotypic experiments and qRT-PCR validation experiments included at least three independent biological replicates. Data are presented as mean ± standard deviation (SD). Statistical analysis and graphing were performed using GraphPad Prism 8.0 software. Comparisons between two groups were analyzed using Student’s *t*-test. Comparisons among multiple groups were analyzed using one-way analysis of variance (ANOVA) followed by Tukey’s post hoc test. Differences were considered statistically significant at *p* < 0.05.

## 3. Results

### 3.1. Bioinformatic Analysis of VdPRMT4

**Figure 1:**
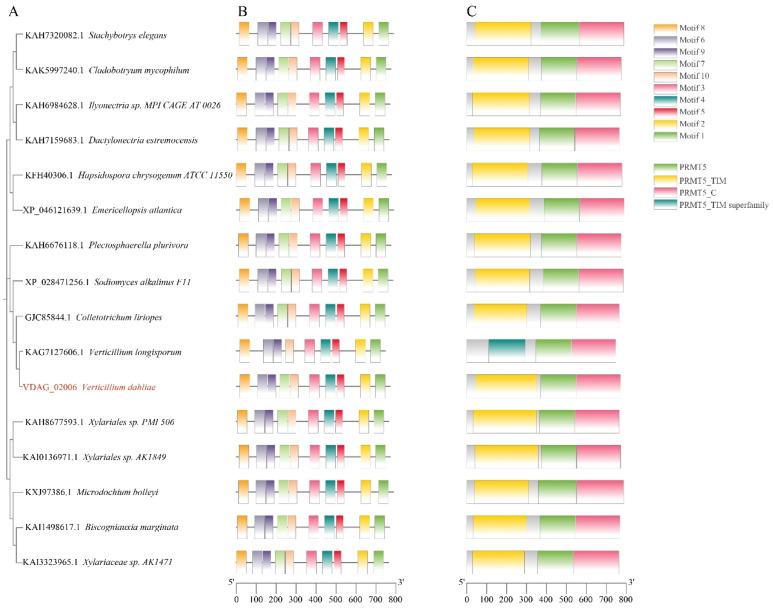
Bioinformatic analysis of *VdPRMT4*. (**A**) Maximum likelihood phylogenetic tree of *VdPRMT4* orthologs (bootstrap = 1000). (**B**) Distribution of conserved motifs in *VdPRMT4* orthologs. Colored boxes represent distinct motifs. (**C**) Predicted conserved domains of *VdPRMT4* orthologs.

To investigate the function of the *VdPRMT4* gene, a systematic bioinformatics analysis was performed. The gene encodes a protein of 771 amino acids with a predicted molecular weight of 85,755.37 Da and an isoelectric point of 5.62. Phylogenetic analysis further classified the PRMTs from different species into three subfamilies and revealed that *VdPRMT4* is most closely related to the KAG7127606.1 protein from *Verticillium longisporum* (Figure 1A). These proteins share a similar motif arrangement in the order Motif8–Motif6–Motif9–Motif7–Motif10–Motif3–Motif4–Motif5–Motif2–Motif1 (Figure 1B). Regarding domain architecture, most proteins contain the PRMT5, PRMT5_TIM, and PRMT5_C domains, whereas the KAG7127606.1 protein possesses PRMT5, PRMT5_TIM superfamily, and PRMT5_C domains (Figure 1C), indicating distinctive features in its domain classification.

### 3.2. V. dahliae Infection Induces the Expression of VdPRMT4

**Figure 2:**
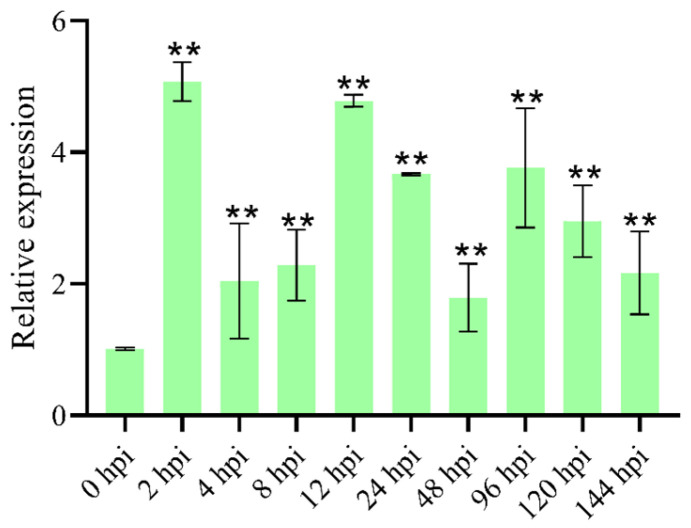
*V. dahliae* infection induces the expression of *VdPRMT4*. Asterisks indicate significant differences in *VdPRMT4* expression at different time points (compared to 0 hpi) as determined by Student’s *t*-test (** *p* < 0.05).

To elucidate the expression dynamics of the *VdPRMT4* gene during *V. dahliae* infection, we quantified its transcript levels at 0, 2, 4, 8, 12, 24, 48, 96, 120, and 144 hpi in *V. dahliae* using qRT-PCR. The results showed that *VdPRMT4* expression was rapidly induced at an early infection stage, reaching the highest peak at 2 hpi. Although the expression level subsequently declined at 4–8 hpi, a second notable peak was observed at 12 hpi, followed by a moderate elevation at 96 hpi (Figure 2). The biphasic expression pattern, with peaks at the early (2 hpi) and mid (12 hpi) infection stages, may reflect distinct roles of *VdPRMT4* during initial host penetration and subsequent vascular colonization. These findings suggest that *VdPRMT4* is significantly upregulated during the host infection by *V. dahliae* and may play an important role in the pathogen’s infection mechanism.

### 3.3. Silencing of VdPRMT4 Reduces Virulence in V. dahliae

**Figure 3:**
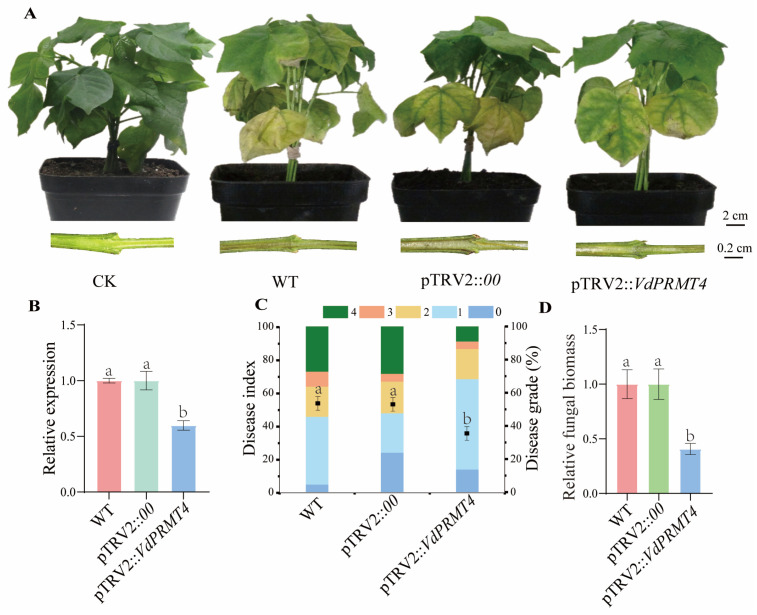
Pathogenicity analysis following HIGS of *VdPRMT4*. (**A**) Relative expression levels of *VdPRMT4* in WT, empty vector control (pTRV2:*00*), and pTRV2:*VdPRMT4* plants as determined by qRT-PCR, assessed once the pTRV2:*GhCLA1* plants displayed the albino phenotype. (**B**) Leaf wilting and vascular browning symptoms in WT, pTRV2:*00*, and pTRV2:*VdPRMT4* plants at 14 days post-inoculation (dpi) with *V. dahliae* strain *Vd*991. (**C**) Disease index of each treatment group two weeks after inoculation, evaluated based on a leaf symptom grading scale. (**D**) Relative fungal biomass in plants from each treatment group at 14 dpi. Data are presented as mean ± SD. Statistical analysis was performed using one-way ANOVA followed by Tukey’s post hoc test. Different lowercase letters indicate statistically significant differences among groups (*p* < 0.05).

To further investigate the biological function of *VdPRMT4*, HIGS was employed for functional validation. After the positive control plants harboring pTRV2:*GhCLA1* displayed the expected albino phenotype (Figure S1), all experimental and control plants were inoculated with a spore suspension (1 × 10^7^ CFU/mL) of the highly virulent *V. dahliae* strain *Vd*991. Disease progression was assessed at 14 dpi. The results showed that both WT and empty vector (pTRV2:*00*) control plants developed severe disease symptoms, including typical wilting, leaf yellowing, and necrosis. In contrast, pTRV2:*VdPRMT4* plants, which exhibited significantly reduced *VdPRMT4* expression, showed markedly attenuated disease symptoms (Figure 3A,B). Consistent with these observations, further statistical analysis of disease indices and measurement of the relative fungal biomass within plant tissues both confirmed that silencing *VdPRMT4* significantly impaired the pathogenicity of *V. dahliae* (Figure 3C,D). Collectively, these results demonstrate that *VdPRMT4* plays a crucial role in the infection and pathogenesis of *V. dahliae*.

### 3.4. Effect of VdPRMT4 Knockout on Mycelial Growth and Sporulation Across Different Carbon Sources

**Figure 4:**
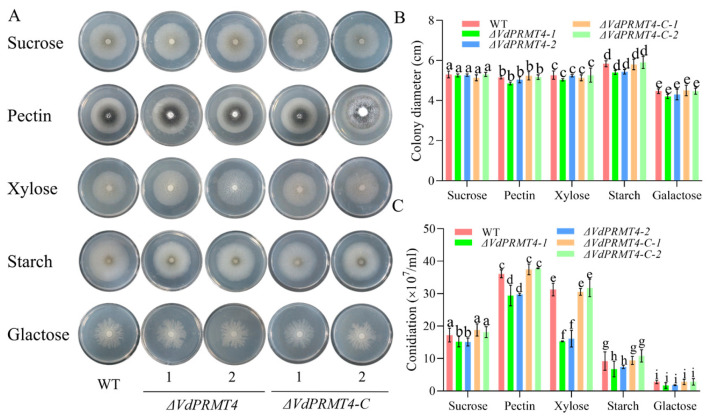
Comparison of growth and morphology among the WT, *ΔVdPRMT4*, and *ΔVdPRMT4-C* on media with different carbon sources. (**A**) Representative colony morphology of each strain after 14 days of cultivation on media containing pectin, galactose, starch, xylose, or sucrose as the sole carbon source. (**B**) Colony diameter of each strain under different carbon source conditions. (**C**) Spore production of each strain under different carbon source conditions. Data are presented as mean ± SD. Statistical analysis was performed using one-way ANOVA followed by Tukey’s post hoc test. Different lowercase letters indicate statistically significant differences among groups (*p* < 0.05).

To investigate whether deletion of *VdPRMT4* affects carbon source utilization in *V. dahliae*, the WT, knockout mutant (*ΔVdPRMT4*), and complemented strains (*ΔVdPRMT4-C*) were cultured on media containing pectin, galactose, starch, xylose, or sucrose as the sole carbon source. After 14 days of cultivation, the growth phenotypes of each strain were systematically assessed. The results showed no significant differences in colony diameter among the three strains across the various carbon sources. However, on all media except galactose, the sporulation capacity of the *ΔVdPRMT4* mutant was significantly reduced compared to that of the WT and complemented strains. The most pronounced decrease in sporulation was observed on xylose-based medium (Figure 4A–C). These findings indicate that while *VdPRMT4* is not essential for the utilization of different carbon sources for radial growth, it plays a crucial regulatory role in conidiation under diverse carbon nutrient conditions.

### 3.5. Effect of VdPRMT4 Knockout on Mycelial Growth and Sporulation Under Various Stress Conditions

**Figure 5:**
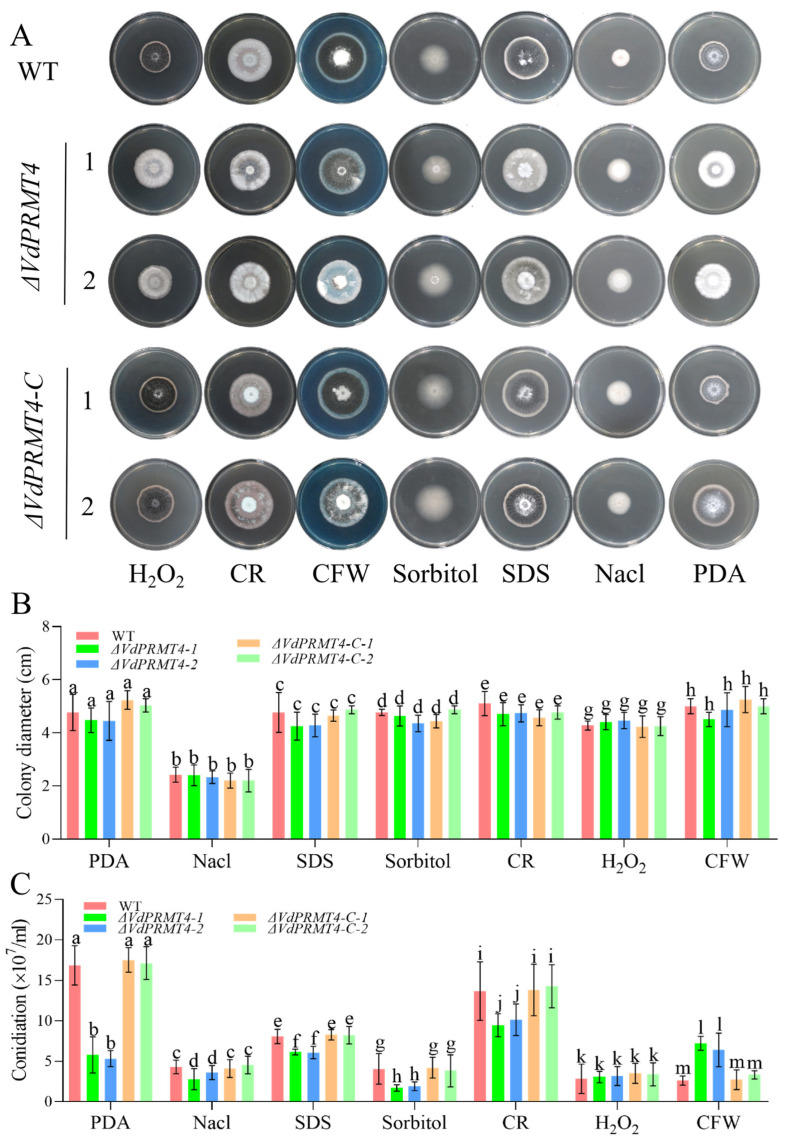
Comparison of growth and morphology among the WT, *ΔVdPRMT4* mutant, and complemented strains (*ΔVdPRMT4-C*) under various stress conditions. (**A**) Representative colony morphology of each strain after 14 days of cultivation on media amended with CR, CFW, SDS, NaCl, or H_2_O_2_. (**B**) Colony diameter of each strain under different stress conditions. (**C**) Spore production of each strain under different stress conditions. Data are presented as mean ± SD. Statistical analysis was performed using one-way ANOVA followed by Tukey’s post hoc test. Different lowercase letters indicate statistically significant differences among groups (*p* < 0.05).

To investigate whether deletion of *VdPRMT4* affects the response of *V. dahliae* to adverse environmental and abiotic stresses, the WT, *ΔVdPRMT4* mutant, and complemented strains (*ΔVdPRMT4-C*) were cultured on media amended with Congo Red (CR, a cell wall perturbant), calcofluor white (CFW, an inhibitor of fungal cell wall synthesis), sodium dodecyl sulfate (SDS, a membrane stress agent), NaCl (high salt), or H_2_O_2_ (oxidative stress). After 14 days of cultivation, the growth phenotypes of each strain were assessed. The results indicated no significant differences in vegetative growth (colony diameter) among the three strains across the various stress conditions. However, when sporulation was evaluated on PDA media supplemented with these stress agents, the *ΔVdPRMT4* mutant exhibited significant alterations compared to those of the WT and complemented strains. The most pronounced reduction in sporulation occurred on standard PDA medium. Intriguingly, on CFW-containing medium, the mutant displayed a higher spore yield than the WT (Figure 5A–C). These findings suggest that while *VdPRMT4* does not markedly affect hyphal tolerance to these stresses, it significantly alters the pathogen’s sporulation capacity under stress conditions, indicating a crucial role for this gene in regulating reproductive adaptation to environmental adversity.

### 3.6. Knockout of VdPRMT4 Significantly Attenuates the Pathogenicity of V. dahliae

**Figure 6:**
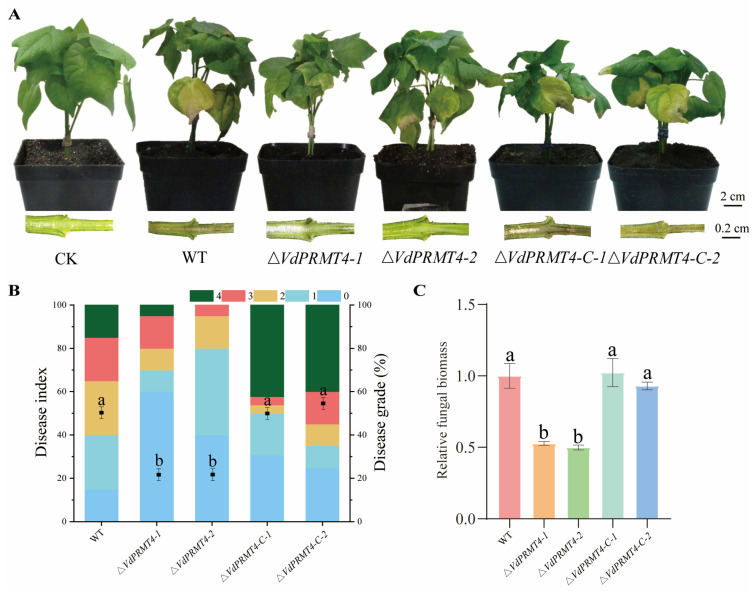
Pathogenicity analysis of cotton plants inoculated with the WT, *ΔVdPRMT4* mutant, and complemented strains (*ΔVdPRMT4-C*). (**A**) Representative disease phenotypes of cotton plants at 14 dpi with each strain. (**B**) Disease index of each treatment group at 14 dpi. (**C**) Relative fungal biomass in the roots of cotton plants from each treatment group at 14 dpi. Data are presented as mean ± SD. Statistical analysis was performed using one-way ANOVA followed by Tukey’s post hoc test. Different lowercase letters indicate statistically significant differences among groups (*p* < 0.05).

To determine the role of *VdPRMT4* in the pathogenicity of *V. dahliae*, cotton seedlings were root-inoculated with spore suspensions (1 × 10^7^ CFU/mL) of *Vd*991, the knockout mutant *ΔVdPRMT4*, the complemented strains *ΔVdPRMT4-C*, or with sterile water as a control. Disease symptoms, disease index, and relative fungal biomass in plant roots were systematically evaluated at 14 dpi. The results showed that plants inoculated with *Vd*991 developed the most severe symptoms, including wilting, leaf yellowing, and necrosis (Figure 6A), with disease severity ratings distributed as grade 4 (15%) and grade 3 (20%). Plants inoculated with the two complemented strains also exhibited severe disease, primarily characterized by grade 4 (42% and 31%, respectively) and grade 3 (3% and 12%, respectively) symptoms. In contrast, plants inoculated with the *ΔVdPRMT4* mutant displayed only mild wilting and yellowing, with one group showing a total of approximately 20% for grades 3 and 4 combined and the other group showing only about 5% (Figure 6B). Quantitative analysis of fungal biomass further demonstrated that the relative biomass of the pathogen remained at approximately 1.0 in both the WT and complemented treatments, whereas it significantly decreased to about 0.5 in the mutant treatment (Figure 6C). Collectively, these results demonstrate that the *ΔVdPRMT4* mutant exhibits significantly reduced pathogenicity, confirming that *VdPRMT4* plays a crucial role in host infection and pathogenic development of *V. dahliae*.

### 3.7. Analysis of Differentially Expressed Genes

Samples from the WT and *ΔVdPRMT4* strains were collected for the construction of six cDNA libraries. Paired-end sequencing was performed on the Illumina NovaSeq 6000 platform. The raw reads obtained for each sample ranged from 58,334,742 to 82,905,724. After stringent quality control, 56,363,220 to 80,311,474 high-quality clean reads were retained for downstream analysis. The GC content of all samples ranged from 58.64% to 59.07%, and the Q20 scores exceeded 99.67%, indicating high sequencing quality. The clean reads were aligned to the reference genome of *V. dahliae*, with unique mapping rates exceeding 90.92% for all samples.

**Figure 7:**
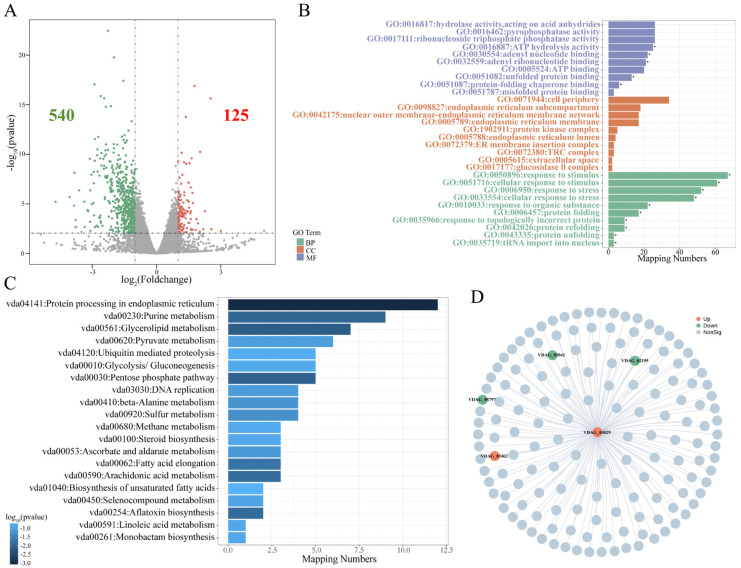
Comparative transcriptome analysis between the WT and the *ΔVdPRMT4*. (**A**) Volcano plot of DEGs. Red dots represent significantly up-regulated genes, green dots represent significantly down-regulated genes, and gray dots represent genes with no significant expression change. (**B**) GO functional enrichment analysis of DEGs. Bar graphs display the most significantly enriched terms in the categories of biological process (BP), cellular component (CC), and molecular function (MF). The length of each bar corresponds to the number of genes enriched in that specific term. (**C**) KEGG pathway enrichment analysis of DEGs. The most significantly enriched metabolic or signaling pathways are shown. (**D**) PPI network analysis of DEGs. In the figure, the asterisk (*) represents statistical significance with *p* < 0.05.

By normalizing read counts and performing differential expression analysis, a total of 665 differentially expressed genes (DEGs) were identified, comprising 125 significantly up-regulated and 540 significantly down-regulated genes (Figure 7A). To explore the potential functional implications of *VdPRMT4*, GO annotation and KEGG pathway enrichment analyses were conducted on the DEGs. GO analysis revealed that the DEGs were significantly enriched in functional categories related to the cell periphery, hydrolase activity, and response to stimulus (Figure 7B). KEGG pathway enrichment analysis further indicated that the deletion of *VdPRMT4* predominantly affected key pathways, including protein processing in the endoplasmic reticulum, purine metabolism, and glycerolipid metabolism (Figure 7C). Further predictive PPI network analysis revealed that the DEGs formed an interaction network centered on the gene *VDAG_00829*, which was significantly up-regulated and is a key gene in the cysteine and methionine metabolism pathway. Network analysis predicted potential interactions between *VDAG_00829* and *VDAG_02195* (from the purine metabolism pathway) and *VDAG_02041* (from the aminoacyl-tRNA biosynthesis pathway), both of which were down-regulated. The network also included two genes of unknown function, *VDAG_08797* and *VDAG_03462*, which showed down-regulated and up-regulated expression patterns, respectively (Figure 7D). Collectively, these transcriptomic results provide exploratory insights, suggesting that *VdPRMT4* modulates a gene interaction network involved in multiple key metabolic pathways.

### 3.8. qRT-PCR Validation of DEGs

**Figure 8:**
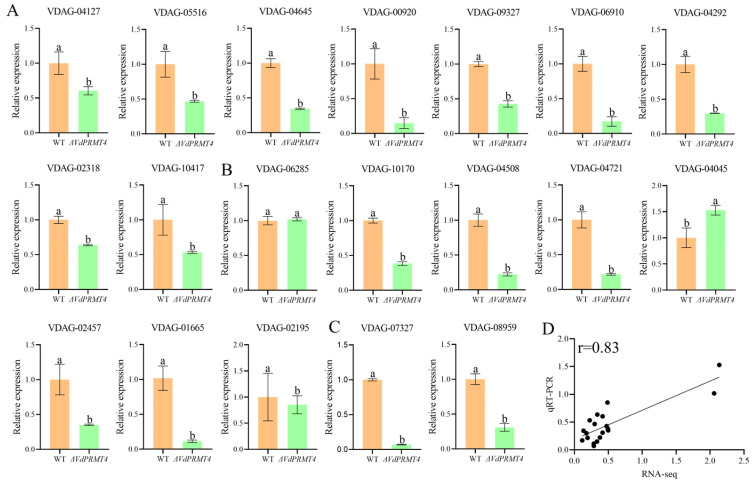
qRT-PCR validation of DEGs in key metabolic pathways. (**A**) Relative expression levels of selected genes in protein processing in the endoplasmic reticulum. (**B**) Relative expression levels of selected genes in purine metabolism. (**C**) Relative expression levels of selected genes in the glycerolipid metabolism pathways. Bars represent mean ± SD (*n* = 3). (**D**) Scatter plot showing the correlation analysis between qRT-PCR validation results and the corresponding gene expression levels from RNA-seq data. The Pearson correlation coefficient (r) is indicated. Different lowercase letters indicate statistically significant differences among groups (*p* < 0.05).

To validate the transcriptome sequencing results and further investigate the molecular mechanism of *VdPRMT4* in cotton resistance to VW, we performed qRT-PCR on a selection of DEGs from three key metabolic pathways previously described: protein processing in the endoplasmic reticulum, purine metabolism, and glycerolipid metabolism. The qRT-PCR results were consistent with the trends observed in the RNA-seq data (Figure 8). Specifically, within the protein processing in the endoplasmic reticulum pathway, the expression levels of genes *VDAG_04127*, *VDAG_05516*, *VDAG_04645*, *VDAG_00920*, *VDAG_09327*, *VDAG_06910*, *VDAG_04292*, *VDAG_02318*, and *VDAG_10417* were all significantly down-regulated under *VdPRMT4* deletion conditions. In the purine metabolism pathway, except for the significant up-regulation of *VDAG_06285*, other genes in this pathway, including *VDAG_10170*, *VDAG_04508*, *VDAG_04721*, *VDAG_04045*, *VDAG_02457*, *VDAG_01665*, and *VDAG_02195,* showed significant down-regulation. Concurrently, the expression of genes *VDAG_07327* and *VDAG_08959* in the glycerolipid metabolism pathway was also significantly decreased. Further statistical analysis revealed a high degree of consistency between the qRT-PCR validation results and the gene expression changes derived from transcriptome sequencing, with a Pearson correlation coefficient of 0.83, strongly supporting the reliability of the transcriptome data in this study.

## 4. Discussion

The pathogenic complexity of cotton VW, caused by *V. dahliae*, extends far beyond traditional virulence factors. This study identifies the protein arginine methyltransferase gene *VdPRMT4* as a key regulator of fungal pathogenicity. The expression level of *VdPRMT4* was rapidly upregulated during the early stage of *V dahliae* infection in cotton, and both HIGS-mediated silencing and targeted gene knockout of *VdPRMT4* significantly attenuated disease symptoms and reduced fungal biomass in planta, demonstrating its essential role in virulence. The critical function of PRMTs in fungal pathogenicity has been increasingly recognized across different species. In *Fusarium graminearum*, deletion of the PRMT homolog AMT1 significantly reduced pathogenicity on wheat heads [28]. In *Magnaporthe oryzae*, PRMT family members, including *MoPRMT4*, *MoPRMT5*, and *MoHMT1,* regulate conidiation, appressorium formation, and infectious growth [29,30]. In *Ustilago maydis* and *Sporisorium reilianum*, double deletion of PRMT1 and PRMT4 resulted in avirulent, non-mating strains, highlighting the essential role of arginine methylation in fungal pathogenesis [31]. In *V. dahliae* itself, the PRMT family member *VdPRMT1* has been reported to affect vegetative growth, sporulation, and pathogenicity on cotton [15]. The loss of *VdPRMT4* severely impaired *V. dahliae* pathogenicity on cotton without affecting basal hyphal growth, consistent with these reports and confirming that arginine methyltransferases are conserved regulators required for full fungal virulence.

Phenotypic analysis of the *ΔVdPRMT4* mutant under diverse nutrient and stress conditions revealed that *VdPRMT4* specifically regulates sporulation adaptability. Although VdPRMT4 deletion did not affect radial growth on various carbon sources (pectin, galactose, starch, xylose, or sucrose), it significantly reduced spore production on all tested carbon sources except galactose, with the most pronounced reduction on xylose. Similarly, under abiotic stresses including cell wall perturbants (Congo Red, calcofluor white), membrane stress (SDS), high salt (NaCl), and oxidative stress (H_2_O_2_), the mutant exhibited normal hyphal growth but altered sporulation capacity—generally reduced, yet intriguingly increased on CFW-containing medium. Genetic complementation fully restored WT phenotypes, confirming that these defects are specifically attributable to loss of *VdPRMT4*. These findings align with reported roles of PRMTs in other fungi. In *Neurospora crassa*, PRMTs interact with the NDR kinase COT1 to regulate hyphal differentiation under stress conditions [32]. In *Magnaporthe oryzae*, PRMT family members are differentially expressed across developmental stages and are essential for conidiation and stress responses [29,30]. In *Sporisorium reilianum*, deletion of PRMT genes affected mating, growth, and virulence due to down-regulation of cell wall-related genes [31]. In *V. dahliae*, deletion of *VdPRMT1* resulted in similar defects in growth and sporulation under various stress conditions [15]. Collectively, these observations suggest that *VdPRMT4* enables *V. dahliae* to fine-tune reproductive strategies in response to environmental cues, thereby maintaining adaptive advantages during host infection.

To elucidate the molecular basis of *VdPRMT4*-mediated pathogenicity, we performed a comparative transcriptome analysis between the WT strain and the *ΔVdPRMT4* mutant. KEGG enrichment analysis revealed that the differentially expressed genes in the *ΔVdPRMT4* mutant were significantly enriched in three key metabolic pathways of *V. dahliae* itself: protein processing in the endoplasmic reticulum (ER), purine metabolism, and glycerolipid metabolism. These pathways are not randomly perturbed but rather represent interconnected cellular processes essential for fungal growth and adaptation. The enrichment of ER protein processing genes suggests that *VdPRMT4* may regulate protein folding, modification, and quality control within the fungal secretory pathway. These processes are essential for the proper maturation and secretion of virulence-related proteins, such as cell wall-degrading enzymes and effectors, which are critical for fungal infection [33]. The enrichment of purine metabolism genes indicates that *VdPRMT4* may be involved in regulating fungal energy homeostasis and nucleotide biosynthesis. Purine nucleotides (e.g., ATP, GTP) are fundamental for DNA replication, RNA transcription, and energy supply during fungal growth, sporulation, and host colonization [24,34]. The enrichment of glycerolipid metabolism genes is particularly significant, as glycerolipids are major structural components of fungal cell membranes and also serve as storage lipids. Proper regulation of glycerolipid metabolism is critical for membrane integrity, spore formation, and stress adaptation in fungal pathogens [35]. The altered expression of these genes upon *VdPRMT4* deletion suggests that this methyltransferase may contribute to fungal pathogenicity by modulating these core metabolic pathways in *V. dahliae*, thereby affecting its growth, development, and infection capacity.

The convergence of these three metabolic pathways points to a coordinated regulatory strategy orchestrated by *VdPRMT4*. How does a fungal methyltransferase achieve such broad effects on fungal metabolism? One possible explanation lies in the regulation of effector gene expression. PRMTs are known epigenetic regulators that control the expression of genes involved in diverse cellular processes, including pathogenesis. In *Sporisorium reilianum*, deletion of *PRMT1* and *PRMT4* led to altered expression of effector genes and complete loss of virulence [30]. In *Ustilago maydis*, transcriptome analysis revealed that epigenetic regulators control the expression of putative effector genes during axenic growth [31]. In *Magnaporthe oryzae,* PRMT family members are differentially expressed during infection and regulate genes required for appressorium formation and invasive growth [29,30]. By analogy, *VdPRMT4* may function as a potential epigenetic regulator that influences the expression of a suite of *V. dahliae* effectors. These effectors, in turn, might target host cellular processes to create a favorable microenvironment for fungal colonization. This multilayered strategy, in which a single epigenetic regulator could control multiple effectors, may represent one possible pathogenic mechanism. The observation that *VdPRMT4* deletion alone leads to significant attenuation of pathogenicity suggests that this gene may play an important role in the pathogenic program.

In conclusion, this study suggests that the arginine methyltransferase *VdPRMT4* may integrate environmental signals to regulate sporulation adaptability under stress and is essential for full pathogenicity of *V. dahliae* on cotton. Transcriptome analysis suggests that *VdPRMT4* influences key metabolic pathways of *V. dahliae*, including protein processing in the endoplasmic reticulum, purine metabolism, and glycerolipid metabolism, potentially through effects on the expression of fungal effector genes. These findings provide additional insights into arginine methyltransferase functions in fungal-plant interactions and suggest *VdPRMT4* as a potential target for developing novel strategies to control VW.

## 5. Conclusions

This study investigates the critical role of the protein arginine methyltransferase gene *VdPRMT4* in the pathogenicity of *V. dahliae*. The results show that *VdPRMT4* expression is markedly up-regulated during the early stages of infection. Although deletion of *VdPRMT4* does not affect basal hyphal growth, it severely impairs the sporulation adaptability of the pathogen under stress conditions and significantly attenuates its virulence on cotton. Comparative transcriptome analysis conducted on PDA medium showed that deletion of *VdPRMT4* led to differential expression of genes involved in core pathways within *V. dahliae*, including protein processing in the endoplasmic reticulum, purine metabolism, and glycerolipid metabolism. This study provides initial evidence suggesting a role of *VdPRMT4* in the pathogenic development of *V. dahliae*, thereby contributing to our understanding of phytopathogenic fungi and suggesting a potential target for the development of novel green control strategies directed against key epigenetic enzymes in this pathogen.

## Data Availability

The data that support the findings of this study are available from the corresponding author upon reasonable request. The raw RNA-seq data generated in this study have been deposited in the NCBI Sequence Read Archive (SRA) and are publicly available under the BioProject accession numberPRJNA1451484.

## Supplementary Materials

The following supporting information can be downloaded at https://www.mdpi.com/article/10.3390/jof12050369/s1. Table S1. Primers used in this study. Figure S1. Phenotype of plants inoculated with the pTRV2::*GhCLA1* vector (showing albino symptoms) after two weeks.
