# Supplementary material for: The Methyltransferase VdPRMT4 Regulates Verticillium dahliae via Regulation of Primary Metabolic Processes
Source: J Fungi (Basel). 2026 May 16;12(5):369. doi: 10.3390/jof12050369 (PMC13208589; doi:10.3390/jof12050369)
Supplement: Supplementary file 1 [file jof-12-00369-s001.zip › jof-4241274-supplementary.pdf]

Table S1. Primers used in this study.

primer names	primer sequences
qRT-Vdactin-F	GGCTTCCTCAAGGTCGGCTATG
qRT-Vdactin-R	GCTGCATGTCATCCCACTTCTTC
VdrDNA-qPCR-F	CCGCCGGTCCATCAGTCTCTCTGTTTATAC
VdrDNA-qPCR-R	CGCCTGCGGGACTCCGATGCGAGCTGTAAC
GhUbq-qPCR-F	AGCTCGGATACGATTGATAACG
GhUbq-qPCR-R	GAAGACGAAGAACAAGGGGAAG
ATMT-PRMT4-1F	gagctcgctgagggtttaattaaGGTGGGCAAATGCCAGTGGTGG
ATMT-PRMT4-1R	gcccgctgaggacttaattaaCCATCTTGATGTCGGGGCTGTCCG
ATMT-PRMT4-2F	cgactagtgctgaggcattaattaaCGAGGCACTCCGGGGAGAGC
ATMT-PRMT4-2R	gaagcttgctgaggtcttaattaaCGTGGGACTCATGGCACGTCATTCG
H-PRMT4-F	gtgagtaaggttaccgaattcCAGCCACTTTCGGACAACCTT
H-PRMT4-R	cgtgagctcggtaccggatccAGGCACGCATGTCTGTCTTGA
MC-4-F	gaattctgccaccttggatccATGGCCGACAACGACAACTACT
MC-4-R	gcccttgctcaccatgtcgacCATCAGGCATGCGACCTTG
q04127-F	ACTTCTTTCCCCCGGTCAAC
q04127-R	TCTCAAAGGGAGCCTCCTCA
q05516-F	GTTGGCCTGGATCTGTCTCC
q05516-R	GCAGCGACACAAGAGTAGGT
q00920-F	ATGAAGAACGACGACACGCT
q00920-R	CGTCAGCGCCAAACATTTCA
q09327-F	TGCTGAGGACAACGATGCTT
q09327-R	TCTCTTCGTGTTGGTGGAGC
q04292-F	TACCACCCTGACAAGAACGC
q04292-R	TGTGAGTGTGGTGGATGGTG
q06285-F	TGTTCGCGGCAAAACTGATG
q06285-R	CCTCGGTTGCCTTCTTGGAT
q10170-F	TTGACGGATCGGTGGGTTTG
q10170-R	GCCAGATGCTCCGTATACCC
q04508-F	CGGGCTAAGAAACTGACGGT
q04508-R	TTGATCTCGAGGCGCTTGTT
q04721-F	CCACCATTGTCAAGAACGCC
q04721-R	TTGTTGGTGAGGTGGATGGG
q04045-F	TTCTTCACACACGCCTCTCC
q04045-R	TGCCGTTTGAAGTGCCATTG
q02457-F	CAGCCTTACCCTGACTGAGC
q02457-R	GTCGTCACGAAGATCACGGA
q01665-F	TCGCCCCTCTCGTTCTTTTC
q01665-R	ATCTTGATGACCTTGGCGCA
q02195-F	GACATGCCCCTGAACGCTAT
q02195-R	CCAGACTCGATGAGGAAGCC
q04645-F	CGCTCGACAAGATCCGCTAT
q04645-R	TGCTTGGAGATGACGGTGAC

q07327-F	GAGCTAGCCAACGACACAGT
q07327-R	ACTGTACAAGCCGTGAGAGC
q06910-F	GCCTCGAGTCTTACGCCTAC
q06910-R	GGTGAGGGTGGTCTTGTCAG
q02318-F	CCCCGTCATTCCTGTCTCAG
q02318-R	CCTCTCAGCCCATGCTACAC
q10417-F	TCTCTGGGCGTCACTATGGA
q10417-R	GCGAAAGGCCAAACTTGAGG
q08959-F	TCGCCCAGTGAGATCAGAGA
q08959-R	TGTCAGAACGTAGCGGAACC

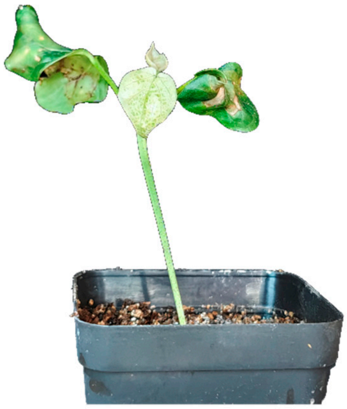

Figure S1. Phenotype of plants inoculated with the pTRV2::*GhCLA1* vector (showing albino symptoms) after two weeks.
